# Correction: A system for tracking whisker kinematics and whisker shape in three dimensions

**DOI:** 10.1371/journal.pcbi.1008723

**Published:** 2021-02-10

**Authors:** Rasmus S. Petersen, Andrea Colins Rodriguez, Mathew H. Evans, Dario Campagner, Michaela S. E. Loft

There is an error in Eq. 13. The denominator of Eq. 13 should be the magnitude of the first derivative of b(s) cubed (|db(s)/ds|^3) instead of the magnitude of the third derivative of b(s) (|d^3b(s)/ds^3|).
