# Supplementary material for: A system for tracking whisker kinematics and whisker shape in three dimensions
Source: PLoS Comput Biol. 2020 Jan 24;16(1):e1007402. doi: 10.1371/journal.pcbi.1007402 (PMC7028309; doi:10.1371/journal.pcbi.1007402)
Supplement: Attachment — Submitted filename: PCOMPBIOL-D-19-01570-review.docx [file pcbi.1007402.s005.docx]

Review Comments

This is a well written paper documenting a new method for tracking whiskers. The authors provide plenty of key technical detail on the importance of this technique, especially for measuring in 3D space. However I believe it needs major revisions with regards to the structure of the paper and its conclusion. I also have minor comments on the paper itself, where I think some details could be clearer to make the paper stronger. I have listed these below with regards, to major concerns and then minor.

Major:

The conclusion for this paper is very weak. It does not express what has been accomplished in the paper and the justification for the need for tracking whiskers in 3D. These points maybe brought up in the discussion, but need to be addressed in a conclusion section. The conclusion section needs to be revised.

The structure of the paper itself, seems to be at odds with itself. It begins with an introduction, results, discussion, and then with discussing the methods. It would be best to revise the structure, so that the methods are presented in details before the results and discussion. This allows the reader to understand the reasoning behind the study, how it is implemented before seeing the results.

**Minor:**

P2 - “Quantification of behaviour is essential for systems neuroscience” This sentence seems out of place with the rest of the abstract and detracts from the rest of the abstract.

P2 - “Here, we developed a high-speed imaging system that measures whisker movements simultaneously from two vantage points” – the phrasing suggests that you developed a new system to track from two separate viewpoints, but in essence is a stereo pair. This in itself isn’t novel. I would suggest rewriting the abstract to focus on the algorithm that tracks whiskers in 3D space and the methods and software you developed around it.

P2 - “The great ethologist Niko Tinbergen described a crucial challenge in biology to measure the total movements made by the intact animal” – This needs a reference.

P3 - “powerful machine vision methods”– This oddly phrased, I would suggest to remove the powerful in this line. It is a machine vision method, not something powerful.

P3 - “Whisker movement is 3D” This seems to be redundant as you explain this in detail in the next sentence. It is not needed in the paper.

P5 – “The degree of match was quantified by a cost function (Methods, Equation 4)” – The algorithm should be explained in more detail in this section as it is integral to the tracking algorithm. It needs to be presented when it is first introduced.

P5 – “Since whiskers imaged as black (low pixel intensity) and background as white (high pixel intensity), the average image intensity along a whisker was low whereas that over a background region was high.” - This sentence is very confusing, by mentioning average image intensity, is that for the entire image? Or is it for the mean intensity for the whiskers? Did you apply any image processing to the image to threshold the image? This needs to be explained clearer for the reader.

P6 - “Fig 4C shows a 12 ms” - The paper switches frequently between the use of ms or frames. Can this be made more consistent in the paper, such as the explaining videos are recorded at 1000fps, and that a frame is the equivalent of 1ms.

P8 – “a rigid disk with curvature comparable to that of a typical mouse whisker ((a 13 mm diameter, glass coverslip), imaged it as described above and tracked its edge (Fig 7A)”. I found this line to be confusing, as it is hard to imagine, how tracking the edge of a rigid disk relates to whiskers. I suggest possibly adding an image of the device, making it clearer to the reader.

P10 – “to be measured with millisecond precision, during both free whisking and whisker-object contact” - The wording is very confusing, as time is not related to precision. Do you mean to say tracking on a frame by frame basis or millimetre accuracy?

P10 – “linear CCD array” - what does CCD refer too?

P10 – “The advance here is an automatic method able to extract a full 3D description of both whisker kinematics and whisker shape” This line is contradicted by the explanation in the methods discussing the tracking pipeline. You explain that a user selects the Bezier points on the whiskers via a GUI and that the user can push spline points back onto the whisker if they come off. This does not fit with an automatic approach as it requires input from a user. It seems to be either a semi-automated approach or a user guided approach. This needs to be addressed through the paper.

P11 – “whisker tracking with head-body tracking (30–33)” - The list of whisker trackers is incomplete (see eg Campagner, 2018; Neuroscience, Gillespie et al; Neuroscience).

P13 – “Mice (C57; males; 6 weeks at time of implant)” In the setup for the experiments, there are no mention of handlers, or the handlers sex etc. These factors are known to influence studies somewhat , yet are not explained in this study. Would these factors impact on your results you received?

P14 – “Since the imaging was done with telecentric lenses, the mappings from 3D to the two image planes were described as orthogonal projections” – Is it possible to explain what telecentric lenses and how they differentiate from normal lenses and how it eliminates the errors that are common in stereo camera calibration.

P15 - “To determine the mapping from 3D to the vertical image plane (𝐕 and 𝐯), we performed the following calibration procedure.” For calibrating stereo pairs, a checkerboard or circle grid is normally used to calibrate two cameras. Can you explain why you used your method over a checkerboard method? Also is it possible to reproduce your calibration method?

P15 – “Using Equation 2, 𝐕 and 𝐯 were then estimated by linear regression.” Are you able to report the error of using linear regression to estimate V and v?
