# Supplementary material for: A system for tracking whisker kinematics and whisker shape in three dimensions
Source: PLoS Comput Biol. 2020 Jan 24;16(1):e1007402. doi: 10.1371/journal.pcbi.1007402 (PMC7028309; doi:10.1371/journal.pcbi.1007402)
Supplement: Attachment — Submitted filename: PCOMPBIOL_reviewers_comments_final.docx [file pcbi.1007402.s006.docx]

Prof R S Petersen

Division of Neuroscience

Faculty of Biology, Medicine & Health

University of Manchester

Stopford Building

Oxford Road

Manchester M13 9PT

UK

+44 161 306 5921

r.petersen@manchester.ac.uk

25^th^ November 2019

Dear Dr Grant and Prof Gershman,

We greatly appreciate the positive feedback on our manuscript and the opportunity to resubmit a revised version to address minor corrections. We thank the editors and the reviewers for their constructive comments. We have revised the manuscript to address the reviewers concerns. Please find below a point-by-point reply. The new version of the manuscript with tracked changes is included in the resubmission.

Yours sincerely,

Rasmus Petersen (On behalf of the authors)

**Reviewer #1:**

General comments:

Seems like a very nice method, nice write up too. Would like to hear a little more about applicability, maybe, to make it as useful as possible to other experimenters.

Thank you. We have added text to discussion concerning the applicability of our method to an existing 2D set-up and to other data (lines 311, 324).

Specific comments:

The authors use feed-forward tracking rather than full-sequence (forward-backwards) tracking. Is there any comment worth making here, trade-off of algorithmic or computational complexity vs performance?

Our GUI allows tracking to be performed either forwards or backwards in time (we have added this point to the text at line 461) but, as the reviewer states, not for both to be combined. Given that performance of the tracker is already good, we suspect that a forward-backward approach would yield limited improvement but at substantial computational cost. We lack data to substantiate this point, however, so prefer not to speculate in the text.

Fig 4 - nice result. Is it cherry-picked, given the discussion in lines 170-178?

Thank you: the sequence does include the complication of whisker overlaps. This is now stated out in the text (line 196):

l208 "In particular, there is a linear relationship between whisker curvature and bending moment." - is this true in general? E.g. for a tapered whisker model? For large bends? Citation?

This relationship applies during quasi-static whisker-object bending. The sentence was imprecise and we thank the reviewer for drawing attention to it, and have corrected the text (line 234):

Why did the authors choose three control points? Was this because this is the minimum number required to measure a bending moment? Or some other reason? l105 "is well-approximated by" is subjective, if practical.

The aim of our algorithm is to track the basal segment of a whisker, which is known from previous research to be well-approximated, during quasi-static whisker object bending and whisking in air, by a quadratic curve (Quist and Hartmann, 2012; Pammer *et al.*, 2013). Since tracking noise increases with the number of parameters that need to be fitted, we used the minimum number of control points sufficient to describe quadratic curve segments. We have amended the text to clarify (line 128).

Fig 7 I got confused about there being only one "horizontal curvature" trace per trial in the lower panel. Worth saying "kv not shown for clarity" (that's right, is it?)?

This is correct and we have changed the legend to Figure 7 to make the point.

Fig 7D is tough to read - is that a polar plot with a false radial origin? Would this be easier to read as a Cartesian plot, perhaps? The actual data, rather than the axes/annotations, is rather cramped.

Fig 7F - I can see why this is plotted polar, because it's periodic, but it's still quite tough to read, I guess because it's a bit cramped, again. Moving things around a bit so that the actual plot lines are a lot larger might help, I think. If 7D is intended to marry up with this one, and that's why it's polar, then same comment there - could be reorganised to use the available space better.

We changed these plots (Figures 7D and 7E) to make them less cramped and to use larger plot lines/symbols. We did try using a Cartesian plot but, since the plots are of an angular variable, we found polar plots to be more effective.

l260 "In this way, 3D imaging permits more accurate measurement of mechanical forces acting on whiskers." Certainly seems that way, good stuff.

Thank you.

l293 "Use of additional cameras provides a potential way to reduce error rates further." Can the authors make a statement on the applicability of the current method to 3+ cameras?

Since each camera makes an additive contribution to the cost function (Equation 4), this is straightforward in principle. We have amended the text of discussion to make the point (line 331).

Perhaps a little more discussion on applicability? Computational load? Equipment requirements? Obstacles to running this online?

We have revised the text of line 324 to comment on applicability to 2D set-ups. The computational load of the software is such that it requires only a standard workstation (e.g., Core i7, 16 GB RAM). This is now stated in Methods (line 370). The main obstacle to running the code on-line is that the code updates the user’s display to show the images and tracking results after each frame.

**Reviewer #2:**

This is a well written paper documenting a new method for tracking whiskers. The authors provide plenty of key technical detail on the importance of this technique, especially for measuring in 3D space. However I believe it needs major revisions with regards to the structure of the paper and its conclusion. I also have minor comments on the paper itself, where I think some details could be clearer to make the paper stronger. I have listed these below with regards, to major concerns and then minor.

**Major:**

The conclusion for this paper is very weak. It does not express what has been accomplished in the paper and the justification for the need for tracking whiskers in 3D. These points maybe brought up in the discussion, but need to be addressed in a conclusion section. The conclusion section needs to be revised.

The conclusion has been revised to incorporate these points (lines 339)

The structure of the paper itself, seems to be at odds with itself. It begins with an introduction, results, discussion, and then with discussing the methods. It would be best to revise the structure, so that the methods are presented in details before the results and discussion. This allows the reader to understand the reasoning behind the study, how it is implemented before seeing the results.

We appreciate the point, but please note that we structured the paper to comply with author guidelines for PLOS Comp Biol, which stipulate “Introduction-Results-Discussion-Methods” format. However, we have revised the section of Results that deals with the cost function to incorporate more information about this and we hope that this goes some way to alleviating the reviewer’s concern.

**Minor:**

P2 - “Quantification of behaviour is essential for systems neuroscience” This sentence seems out of place with the rest of the abstract and detracts from the rest of the abstract.

This has been rephrased (line 23).

P2 - “Here, we developed a high-speed imaging system that measures whisker movements simultaneously from two vantage points” – the phrasing suggests that you developed a new system to track from two separate viewpoints, but in essence is a stereo pair. This in itself isn’t novel. I would suggest rewriting the abstract to focus on the algorithm that tracks whiskers in 3D space and the methods and software you developed around it.

We have revised the phrasing and changed the abstract to include more detail on the algorithm.

P2 - “The great ethologist Niko Tinbergen described a crucial challenge in biology to measure the total movements made by the intact animal” – This needs a reference.

This has been done (line 37).

P3 - “powerful machine vision methods”– This oddly phrased, I would suggest to remove the powerful in this line. It is a machine vision method, not something powerful.

This has been done (line 52).

P3 - “Whisker movement is 3D” This seems to be redundant as you explain this in detail in the next sentence. It is not needed in the paper

We have revised this sentence to better describe the topic of the paragraph (line 58).

P5 – “The degree of match was quantified by a cost function (Methods, Equation 4)” – The algorithm should be explained in more detail in this section as it is integral to the tracking algorithm. It needs to be presented when it is first introduced.

The Results text has been revised to include the cost function equation and intuitive explanation of it line 142ff).

P5 – “Since whiskers imaged as black (low pixel intensity) and background as white (high pixel intensity), the average image intensity along a whisker was low whereas that over a background region was high.” - This sentence is very confusing, by mentioning average image intensity, is that for the entire image? Or is it for the mean intensity for the whiskers? Did you apply any image processing to the image to threshold the image? This needs to be explained clearer for the reader.

We have rewritten this sentence to clarify and to link it more directly to explanation of the cost function (previous comment). No image thresholding was performed.

P6 - “Fig 4C shows a 12 ms” - The paper switches frequently between the use of ms or frames. Can this be made more consistent in the paper, such as the explaining videos are recorded at 1000fps, and that a frame is the equivalent of 1 ms.

The Results states the frame rate (line 100). To improve clarity, we now also state frame rate in the relevant figure legends (Figs 4 and 7) and rephrased these figure legends in terms of frames.

P8 – “a rigid disk with curvature comparable to that of a typical mouse whisker ((a 13 mm diameter, glass coverslip), imaged it as described above and tracked its edge (Fig 7A)”. I found this line to be confusing, as it is hard to imagine, how tracking the edge of a rigid disk relates to whiskers. I suggest possibly adding an image of the device, making it clearer to the reader.

A segment of the edge of the disk is, like a whisker, a 3D curve segment. Images of the disk are shown in Fig 7A and we have amended the text to clarify that it is the edge of the disk that has curvature similar to that of a whisker (line 252).

P10 – “to be measured with millisecond precision, during both free whisking and whisker-object contact” - The wording is very confusing, as time is not related to precision. Do you mean to say tracking on a frame by frame basis or millimetre accuracy?

We have revised the text to specify that it is millisecond frame rate (line 306)

P10 – “linear CCD array” - what does CCD refer too?

Charge coupled device. This is now defined (line 68).

P10 – “The advance here is an automatic method able to extract a full 3D description of both whisker kinematics and whisker shape” This line is contradicted by the explanation in the methods discussing the tracking pipeline. You explain that a user selects the Bezier points on the whiskers via a GUI and that the user can push spline points back onto the whisker if they come off. This does not fit with an automatic approach as it requires input from a user. It seems to be either a semi-automated approach or a user guided approach. This needs to be addressed through the paper.

Thanks for the comment – we now see that the original submission was not fully clear. Our core algorithm (after the initialisation step) requires no manual intervention and is automatic. However, unlike some previous whisker trackers, our software includes a GUI tool which provides users with the optional opportunity to manually curate tracking results. This tool allows errors in automatic tracking to be corrected and thus enables both error rate to be lowered and extends the scope of the system to more challenging data. We have amended the text to clarify this (line 27, line 199, line 209, line 311).

P11 – “whisker tracking with head-body tracking (30–33)” - The list of whisker trackers is incomplete (see eg Campagner, 2018; Neuroscience, Gillespie et al; Neuroscience).

This has been done (line 338).

P13 – “Mice (C57; males; 6 weeks at time of implant)” In the setup for the experiments, there are no mention of handlers, or the handlers sex etc. These factors are known to influence studies somewhat , yet are not explained in this study. Would these factors impact on your results you received?

It is possible that these factors might have some influence one whisker kinematics but we are not aware of any reason why they would impact on the applicability or success of our algorithm.

P14 – “Since the imaging was done with telecentric lenses, the mappings from 3D to the two image planes were described as orthogonal projections” – Is it possible to explain what telecentric lenses and how they differentiate from normal lenses and how it eliminates the errors that are common in stereo camera calibration.

Explanation of this has been added (line 424ff).

P15 - “To determine the mapping from 3D to the vertical image plane (𝐕 and 𝐯), we performed the following calibration procedure.” For calibrating stereo pairs, a checkerboard or circle grid is normally used to calibrate two cameras. Can you explain why you used your method over a checkerboard method? Also is it possible to reproduce your calibration method?

We didn’t try the checkerboard approach but it would likely also be effective. The description of our calibration method in the text should allow for it to be reproduced.

P15 – “Using Equation 2, 𝐕 and 𝐯 were then estimated by linear regression.” Are you able to report the error of using linear regression to estimate V and v?

Thanks for this suggestion. We performed new analysis to check this, finding that the ratio of residual variance to total variance was <0.1%. This is now reported in the text (line 441).

**Reviewer #3:**

The study of Petersen et al. describes a novel combination of known computer-vision technologies, and an impressive auto-tracker, to the specific problem of tracking whisker shape in 3D. While potentially suitable for publication as a Methods paper in PLoS CB, there are a few concerns.

1. As it currently stands, the manuscript gives the impression that the 3D merging works because Bezier curves were used. In fact, as indicated in Methods, the 3D merging approach is viable because the authors performed a point-to-point calibration between the cameras using two pins mounted on a motor.

It will likely be helpful to readers to explain the basic problem more clearly: in order to 3D merge, it is necessary either to ensure that several points along the whisker length can be directly matched between the two cameras, or to perform pixel-to-pixel calibration between cameras. The first approach was used by Knutsen et al., 2008, who placed dye at known points along the whisker length. The second approach was used by Huet et al., 2015 and is used in the present work. This information would help readers better compare technical approaches between studies, and would highlight the novelty of the present work, which lies in automating the approach for multiple whiskers and in making the code publicly available.

We take the point and have changed the text accordingly. In the revised text, we now explain the 3D merging problem, do so before the Bezier approach is introduced, and have changed the relevant subsection heading to better reflect the above (line 98, line 111ff).

2. Because this is a Methods paper, it would be helpful for the introduction to be more clear that the approach is currently limited to the head fixed animal, to a single row of whiskers, and for very specific locations of the cameras.

We have revised the Introduction to make explicit the use of a head-fixed mice prep and the positioning of cameras to minimise occlusion (line 77). Please note that the tracker can work for multiple rows (example of Fig4 includes whiskers on C,D and E rows).

3. As currently written the manuscript risks misleading the field about the relationship between whisker curvature and the mechanical signals at the whisker base. Specifically, the manuscript states: “… there is a linear relationship between whisker curvature and bending moment,” and that several studies have computed bending moment based on curvature.

But the linear relationship between curvature and bending moment holds only in quasistatic conditions. During non-contact (“free-air”) whisking and during collisions, the quasistatic approximation does not hold. To compute mechanical signals at the whisker base under these conditions requires a dynamic model of the whisker. Notably, computing whisker dynamics requires (among many other things) knowledge of the entire whisker shape all the way to the tip, not just the proximal portion.

The manuscript should add a paragraph explicitly warning the reader that even though the curvature can be computed during non-contact whisking, during texture exploration, and immediately after collision with an object, this curvature is not a good proxy for any particular mechanical signal at the whisker base. The curvature is a good proxy for bending moment only during pure bending, as the whisker deflects against an object, after vibrations from the initial collision have damped out.

Note that curvature might still correlate with a variety of neural signals, but it is not uniquely related to the mechanical signals at the whisker base (except during pure bending).

We take the point and have amended the text to include the warning suggested by the reviewer (lines 234ff).

Minor:

The sentence: “Moreover, estimates of whisker-object bending force (‘bending moment’) obtained by imaging apparent curvature of a whisker…” makes it sound as though force and bending moment are synonymous. They are very different. Also the idea of “bending force” is not clear.

This has been corrected (line 70).

The sentence: “This is significant, since bending moment is the primary driver of contact-related mechanotransduction (23–25).” should be changed to “one of the primary drivers” as the axial (longitudinal) force may also play a significant role (Stuttgen and Schwartz). In addition, the transverse forces and twisting moment could also be important – no study has yet shown otherwise.

This has been corrected (line 72).

The sentence: “First, during whisker-object contact, whiskers bend and the associated mechanical force/moment drives mechanoreceptors” should be changed to forces and moments to indicate that they are distinct quantities and both plural.

This has been done (line 104).
